# DUBs in Alzheimer’s disease: mechanisms and therapeutic implications

**DOI:** 10.1038/s41420-024-02237-3

**Published:** 2024-11-20

**Authors:** Biying Qin, Xiaodong Chen, Feng Wang, Yanfeng Wang

**Affiliations:** 1 Key Laboratory of Molecular Medicine and Biotherapy, School of Life Science, Beijing Institute of Technology, Beijing, China; 2 Tangshan Research Institute, Beijing Institute of Technology, Tangshan, Hebei, China; 3 Advanced Technology Research Institute, Beijing Institute of Technology, Jinan, Shandong China

**Keywords:** Health sciences, Target identification, Alzheimer's disease

## Abstract

Alzheimer’s disease (AD) is a prevalent neurodegenerative disorder characterized by the accumulation of amyloid β protein (Aβ) and the hyper-phosphorylation of the microtubule-associated protein Tau. The ubiquitin-proteasome system (UPS) plays a pivotal role in determining the fate of proteins, and its dysregulation can contribute to the buildup of Aβ and Tau. Deubiquitinating enzymes (DUBs), working in conjunction with activating enzymes (E1), ubiquitin-conjugating enzymes (E2), and ubiquitin ligases (E3), actively maintain the delicate balance of protein homeostasis. DUBs specifically remove ubiquitin tags from proteins marked for degradation, thereby averting their proteasomal breakdown. Several DUBs have demonstrated their capacity to regulate the levels of Aβ and Tau by modulating their degree of ubiquitination, underscoring their potential as therapeutic targets for AD. In this context, we present a comprehensive review of AD-associated DUBs and elucidate their physiological roles. Moreover, we delve into the current advancements in developing inhibitors targeting these DUBs, including the determination of cocrystal structures with their respective targets. Additionally, we assess the therapeutic efficacy of these inhibitors in AD, aiming to establish a theoretical foundation for future AD treatments.

## Facts

- The accumulation of Aβ and Tau in the central nervous system are considered to be the two major causes of AD.
- Some deubiquitinating enzymes have been shown to be able to counteract the adverse effects of Aβ or Tau protein degradation.
- DUBs have become one of the important cellular targets for AD treatment.

## Open questions

- Are there other effective strategies for treating AD besides developing pharmacological inhibitors targeting DUB?
- Whether DUBs in neurons regulate the same signaling pathways as DUBs in other cells in the body?

## Introduction

**Fig. 1:**
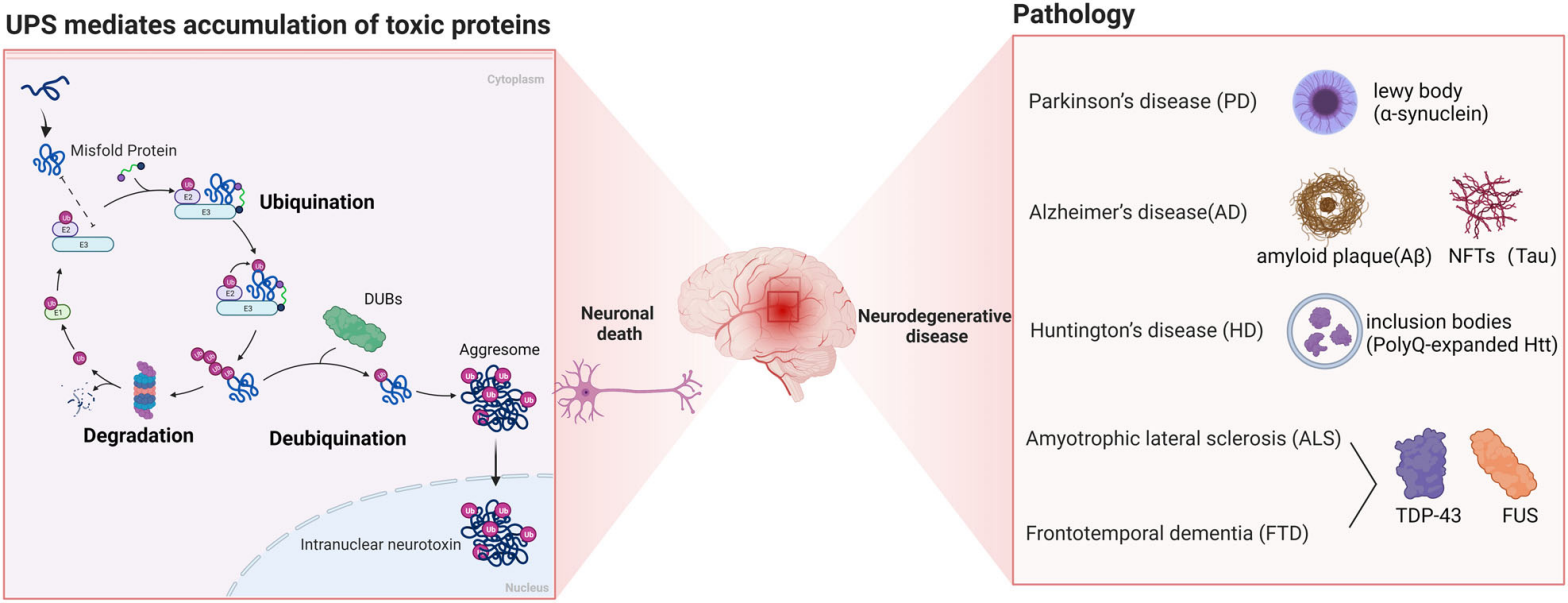
The Link between the UPS and NDs. The UPS represents a key process for protein degradation, involving ubiquitinating enzymes (E1, E2, and E3) and the 26S proteasome. The UPS can be divided into two main steps: ubiquitination and protein degradation. During the ubiquitination process, a multistep cascade reaction occurs, starting with E1 enzymes utilizing ATP hydrolysis to generate a thioester bond between the C-terminal of ubiquitin and a cysteine residue in the active catalytic site of E1 enzymes. The activated ubiquitin is then transferred to E2 enzymes, forming a thioester bond between the E2 enzymes and ubiquitin. Finally, the charged E2 enzymes work in conjunction with E3 enzymes to transfer the activated ubiquitin to the misfolded protein substrate. The ubiquitin-tagged protein is then directed to the active center of the proteasome for degradation. On the other hand, DUBs play a crucial role in the UPS by removing ubiquitin tags from substrate proteins. In neurodegenerative diseases, the dysregulation of DUBs leads to the accumulation of misfolded proteins, which aggregate and become intracellular neurotoxins. This process ultimately results in neuronal death and the manifestation of various pathological characteristics associated with neurodegenerative diseases. UPS Ubiquitin-Proteasome System, NDs Neurodegenerative Diseases, E1 ubiquitin-activating enzymes, E2 ubiquitin-conjugating enzymes, E3 ubiquitin ligases, DUBs deubiquitinating enzymes.

Neurodegenerative diseases (NDs) such as Alzheimer’s disease (AD), Huntington’s disease (HD), Parkinson’s disease (PD), amyotrophic lateral sclerosis (ALS), and frontotemporal dementia (FTD) are prevalent among elderly individuals and cause significant distress to patients and their families [1]. Although these diseases have distinct pathophysiological characteristics, they share a common feature, which is the accumulation of dysfunctional neurotoxic proteins in the central nervous system (CNS) [2, 3]. For instance, PD is characterized by the presence of Lewy bodies (α-synuclein) [4], HD is an autosomal dominant ND caused by the aberrant expansion of inclusion bodies (polyQ-expanded Htt) [5], ALS and FTD are pathologically characterized by the aggregation of TAR DNA-binding protein 43 (TDP-43) and fused in sarcoma (FUS) (Fig. 1) [6]. Due to this prominent feature, NDs are often referred to as “protein diseases” [7].

AD is the most common neurodegenerative disease. Current research has identified neuroinflammation, autophagy induction, UPS abnormalities, cell death, genetic mutations, oxidative stress, synaptic dysfunction, and neurotransmitter imbalance as key factors contributing to the onset of AD [8]. The accumulation of the amyloid β protein (Aβ) and Tau represent the major hallmarks of AD [9]. In 1984, Glenner & Wong discovered that Aβ is the central component of extracellular amyloid plaques in AD [10]. Two years later, researchers unveiled that the primary component of neurofibrillary tangles (NFTs) is tau protein, and they proposed that the hyperphosphorylation of tau is a major cause of neuronal degeneration in AD [11]. Therefore, the clearance of Aβ and Tau is crucial for AD. Intracellular clearance of neurotoxic proteins predominantly occurs through two protein degradation systems: the ubiquitin-proteasome system (UPS) and the autophagy-lysosomal pathway (ALP). The UPS pathway accounts for 80%-90% of cellular protein degradation [12–14].

In the UPS, ubiquitin conjugation to the substrate occurs through a multistep cascade reaction involving ubiquitin-activating enzymes (E1), ubiquitin-conjugating enzymes (E2), and ubiquitin ligases (E3) (Fig. 1) [7, 15]. Through E1, E2, and E3 enzymes, substrates can be tagged with different types of ubiquitin chains (including M1, K6, K11, K27, K29, K33, K48, and K63), participating in various signaling pathways [16]. Canonical K48-linked polyUb chains are believed to be the principal signal for targeting substrates for degradation by the 26S proteasome, whereas K63-linked chains act in a range of other processes, including protein trafficking, DNA repair, and inflammation. K6 linkages might regulate DNA repair, K11 linkages are primarily involved in the fine-tuning of cell cycle regulation, both K27 and K33 linkages may be assembled by U-box-type E3 ligases during stress response, finally, K29-linked chains may participate in Ub fusion degradation [17]. During protein quality control, soluble misfolded proteins are tagged with polyubiquitin, primarily K48-linked polyubiquitin, for 26S proteasomal degradation. [7, 18–20]

Deubiquitinating enzymes (DUBs) counteract the activity of E3 ligases by removing the ubiquitin chain from target proteins, thereby preventing their degradation and reversing functional changes caused by ubiquitination (Fig. 1) [19, 21]. Over 100 DUBs have been identified, and based on sequence and domain conservation, they can be classified into seven families: ubiquitin-specific peptidases (USPs), Jab1/Mov34/Mpr1 Pad1 N-terminal+ domain proteases (JAMMs), ovarian tumor proteases (OTUs), Machado–Josephin domain proteases (MJDs), ubiquitin C-terminal hydrolases (UCHs), motif interacting with ubiquitin-containing novel DUB family proteases (MINDYs), and zinc finger-containing ubiquitin peptidase 1 (ZUP1) [22, 23].

Several DUBs have been found to inhibit the degradation of Aβ or Tau through deubiquitination. For example, USP9X deubiquitinates K29/33-linked Mark4, promoting the phosphorylation of Tau [24, 25]. While OTUB1 prevents the decomposition of Tau by removing the K48-linked polyubiquitin chain [26]. Additionally, studies have shown that the N-terminus of Aβ is a possible binding site for ubiquitin with Ub-K48 and -K63 chains, and ubiquitination of Aβ by the Ub-K63 chain leads to aggregation and delays the degradation of Aβ [27]. Therefore, it can be speculated that deubiquitinases and these types of ubiquitin chains play a critical role in the degradation of Aβ and Tau in AD.

In this review, we discuss AD-related DUBs and their physiological roles, describe the development of current inhibitors and cocrystal structures of these DUBs, and evaluate the therapeutic effects of these inhibitors in AD. Our aim is to provide a theoretical basis for future AD therapy.

## Physiological role of DUBs in AD

**Fig. 2:**
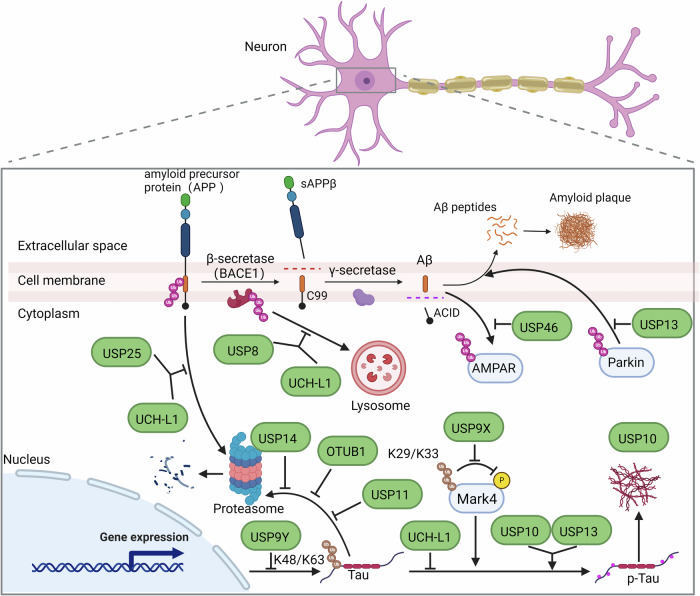
The cellular Role of DUBs in AD. The excessive production and accumulation of Aβ and p-Tau are the main pathological features of AD. In the amyloidogenic pathway, APP is initially cleaved by BACE1 and subsequently cleaved by γ-secretase, leading to the generation of Aβ peptides. The accumulation and aggregation of Aβ peptides contribute to the formation of neurotoxic amyloid plaques. Several DUBs have been implicated in the regulation of key proteins involved in AD pathogenesis. USP8, USP25, USP46, and UCH-L1 play roles in modulating the stability of APP and BACE1, thus influencing the production and accumulation of Aβ. Furthermore, in the context of Tau pathology, p-Tau proteins aggregate to form NFTs. In this process, USP9Y regulates Tau transcription, while USP14 and OTUB1 inhibit Tau degradation mediated by the proteasome. Additionally, USP9X, USP10, USP11, USP13, and UCH-L1 are involved in the regulation of Tau phosphorylation. Aβ amyloid β protein, AD Alzheimer’s disease, APP amyloid precursor protein, BACE1 β-secretase, DUBs deubiquitinating enzymes, NFTs neurofibrillary tangles.

AD, characterized by abnormal aggregation of Aβ and Tau, is a representative neurodegenerative disease [28]. DUBs, as regulatory factors of Aβ and Tau, have emerged as important therapeutic targets (Table 1**) (**Fig. 2). Abnormal expression levels or functional alterations of DUBs have been implicated in the pathogenesis of AD [29].

**Table 1:** The physiological roles of AD-related DUBs.

DUB	Disease relevance	Functions	Refs.
DUBs regulate Aβ			
USP8	Upregulated	Reduces K501 ubiquitination of BACE1	[35]
USP25	Upregulated	Rescues the degradation of APP	[42]
USP46	Downregulated	Reverses Aβ-dependent AMPAR ubiquitination	[34, 45]
DUBs regulate Tau			
USP9 X	Upregulated	Regulates of phosphorylation and expression of Tau	[24, 25]
USP9 Y	Downregulated		
OTUB1	Upregulated	Removes the K48 polyubiquitin chain from Tau	[26]
USP14	Upregulated	Inhibits the degradation of Tau by 26S proteasome	[56, 58]
USP10	Upregulated	Binds and deubiquitinates Tau	[61]
USP11	Upregulated	Deubiquitinates Tau at K281	[63]
DUBs regulate Aβ and Tau			
UCH-L1/3/5	Downregulated	Controls the degradation of APP, BACE1, and Tau	[67, 68, 71]
USP13	Upregulated	Deubiquitinates parkin and Tau	[87, 89, 157]

The roles and pathways of DUBs in the development of AD.

### DUBs regulate Aβ

#### USP8 Depletion Decreases BACE1 and Aβ Levels in H4 human neuroglioma cells

Aβ, a toxic peptide that accumulates in the brains of individuals with AD, is generated by sequential cleavage of APP by β-secretase and γ-secretase [24, 30]. BACE1, the β-secretase in vivo, is the rate-limiting enzyme in Aβ production [31]. Studies have shown that BACE1 can be degraded through the lysosome [32], with K501 ubiquitination serving as a prerequisite for lysosomal pathway-mediated degradation [33]. Ubiquitin-specific peptidase 8 (USP8), an endosome-associated deubiquitinating enzyme, regulates the ubiquitination, trafficking, and lysosomal degradation of several plasma membrane proteins [34]. RNAi-mediated depletion of USP8 reduced levels of both ectopically expressed and endogenous BACE1 by deubiquitinating K501 in H4 human neuroglioma cells [35]. Additionally, USP8 depletion reduces BACE1-mediated APP cleavage and Aβ formation [35]. These findings suggest that targeting USP8 to enhance BACE1 degradation may represent a potential therapeutic strategy for AD.

#### USP25 promotes AD by regulating IL-17 signaling and ER stress

As the major immune cells in the brain, microglia play a key role in maintaining central nervous system (CNS) homeostasis and protecting the brain from infection and injury [6, 7]. Short-term activation of microglia may facilitate debris clearance and tissue repair [36]. However, sustained activation of microglia induces chronic release of proinflammatory cytokines, thereby initiating inflammatory cascades and pathogenic neurotoxic effects in neurodegeneration [36]. The overexpression of the deubiquitinating enzyme USP25, which is encoded by a gene located on chromosome 21, led to microglial activation and induced synaptic and cognitive dysfunction, as observed in trisomy 21-associated AD [37]. USP25 has been identified as a negative regulator of interleukin-17 (IL-17) signaling and inflammatory responses [38]. Additionally, USP25 deficiency suppresses the transcriptional activity of interferon regulatory factors, thereby reducing type I interferon production [39]. In mouse models overexpressing USP25, a reduction in dendritic spine density in hippocampal neurons, microglial activation, and impaired spatial memory are observed [40]. Conversely, USP25 deficiency mitigates excessive microglia-mediated production of proinflammatory cytokines and synaptic elimination [40]. Genetic deletion or pharmacological inhibition of USP25 enhances Aβ-induced synapse phagocytosis, restores microglial homeostasis, and reduces neuroinflammation and cognitive impairment in AD mice, highlighting USP25 as a promising therapeutic target for AD.

Growing evidence indicates that abnormal endoplasmic reticulum (ER) calcium homeostasis may contribute to the pathogenesis of AD [41]. APP is rapidly degraded by the ubiquitin-proteasome system (UPS) in the CHO cell line in response to endoplasmic reticulum (ER) stress, such as calcium ionophore, A23187, resulting in reduced Aβ levels, a major pathological hallmark of AD [37]. Furthermore, decreased levels of USP25 in CHO cells were observed during ER stress. USP25 interacts with APP under steady-state conditions, promoting its stability and leading to the accumulation of misfolded APP. However, this interaction is disrupted under ER stress [42]. Moreover, overexpression of USP25 rescues the A23187-induced degradation of APP, indicating the requirement of USP25 for APP stabilization and the generation of its metabolites, including Aβ40 and Aβ42 [42].

#### USP46 antagonizes Aβ-induced AMPAR ubiquitination in synaptic transmission

AMPA glutamate receptors (AMPARs) play a crucial role in excitatory synaptic transmission and advanced brain function [43]. Ubiquitination of AMPAR is a crucial molecular event leading to its loss and subsequent inhibition of synaptic transmission in AD [44]. Aβ incubation triggers AMPAR ubiquitination, followed by receptor internalization and degradation. Zhang et al [45]. discovered that the expression of AMPAR and its deubiquitinating enzyme, Ubiquitin-specific peptidase 46 (USP46), significantly decreased in Aβ-treated neurons and human brain tissues of AD patients. Conversely, the ubiquitination of AMPAR and the expression of the E3 ligase Nedd4 were increased under these conditions [34]. USP46 is directly involved in Aβ-dependent AMPAR ubiquitination, and its overexpression can partially rescue the decrease in AMPAR levels [45]. Therefore, USP46 may hold great significance in the treatment of AD caused by Aβ accumulation.

### DUBs regulate Tau

#### USP9 promotes Tau phosphorylation in neuronal cells

Gender differences exist in the incidence and phenotypic manifestations of AD, with women being twice as predisposed to AD as men [46]. Additionally, female AD patients exhibit more severe pathology and faster brain atrophy compared to male AD patients [47]. USP9 can be classified into the Y-chromosomal form (USP9Y) and the X-chromosomal form (USP9X), depending on sex [48]. USP9Y is specifically expressed in males and is decreased in AD patients. It inhibits Tau at the transcriptional level [25]. Previous studies have demonstrated that USP9 knockdown significantly reduces Tau activity in cells and zebrafish models. Microtubule affinity-regulating protein 4 (Mark4), a known Tau kinase, exhibits markedly increased expression and interacts with Tau in postmortem AD brains [49]. USP9X facilitates the deubiquitination of Mark4 by removing K29/33-linked ubiquitin chains, promoting AD-related phosphorylation of the microtubule-associated protein Tau [24, 25]. Moreover, USP9X can increase Tau phosphorylation via a second mechanism, by deubiquitinating the protein α-synuclein, which functions as a connecting mediator between the glycogen synthase kinase 3β (GSK3B) and Tau, it has been shown to stimulate Tau phosphorylation via GSK3B in neuronal cells [50]. Hence, USP9 can regulate Tau through both translational and post-translational modifications.

#### OTUB1 prevents Tau degradation in primary neurons

The OTU deubiquitinase ubiquitin aldehyde-binding 1 (OTUB1) is commonly found in amyloid plaques associated with AD [51, 52]. Research has demonstrated that OTUB1 prevents Tau degradation by removing K48 polyubiquitin chains from Tau in both mouse brains and primary neurons [26]. Inhibition of OTUB1 expression impairs Tau degradation, leading to the accumulation of phosphorylated and oligomeric Tau proteins. The deubiquitination of Tau by OTUB1 relies on its typical DUB activity, as the catalytically inactive C91A mutant fails to deubiquitinate Tau [26]. These findings suggest that OTUB1 may contribute to the development of pathological diseases associated with Tau aggregates. OTUB1 inhibitors, primarily being developed for cancer treatment, may provide an effective therapeutic avenue for AD in the future.

#### USP14 inhibits the degradation of Tau by the proteasome

Ubiquitin-specific peptidase 14 (USP14) has been identified as a proteasome-associated DUB, and there is a compensatory increase in USP14 activity accompanies impaired proteasomal proteolysis during aging [53]. USP14 is activated through specific binding to the 26S proteasome and catalyzes the removal of ubiquitin from target proteins before their degradation by the proteasome [54]. USP14 reduces the degradation of pathogenic proteins by the 26S proteasome and is implicated in various neurodegenerative diseases, including AD [55]. As mentioned earlier, abnormal phosphorylation of Tau is observed in AD, and depletion of USP14 enhances the accumulation of p-Tau [56]. The inhibitory effect of USP14 on proteasome degradation depends on its deubiquitinating activity. In HEK293 cells, co-expression of wild-type USP14 with Tau or TDP-43 leads to higher expression levels compared to non-catalytic (C114A) USP14 [57]. Additionally, USP14 is essential for synaptic development and normal function of neuromuscular junctions (NMJs). USP14-deficient mice exhibit motor neuron deficits and developmental defects in NMJs [58]. Therefore, inhibition of USP14 has been proposed as a therapeutic strategy to enhance proteasome function in neurodegenerative diseases [59].

#### USP10 induces Tau aggregation in primary neurons

Recent research suggests that USP10 is involved in Tau phosphorylation. USP10 is upregulated in the brains of AD patients and APP/PS1 mice [60]. In primary cultured neurons, Aβ42 induces USP10 overexpression and promotes binding of Tau to USP10, resulting in a significant decrease in Tau ubiquitination. Consequently, Tau and p-Tau proteins are significantly increased, and p-Tau further aggregates to form neurofibrillary tangles [61]. The use of Tau307-326K and Tau341-378K interference peptides competitively inhibits the binding of Tau to USP10, leading to attenuated Tau hyperphosphorylation and deubiquitination [61]. These results suggest that USP10 plays a key role in mediating Tau aggregation, blocking USP10-induced p-Tau accumulation could be an important therapeutic strategy in the early stage of AD.

#### USP11 is a molecular switch for Tau acetylation and aggregation in iHEK-tau^P301L^ cells

Despite the well-established gender disparity in AD, the underlying mechanism for increased vulnerability in women compared to men remains unclear [62]. Yan et al. addressed this issue by studying in vitro and in vivo models, as well as human AD brain tissue [63]. They found that USP11, which escapes complete X-inactivation, is expressed at higher levels in females due to its X-linked nature. In transfected iHEK-tau^P301L^ cells, X-linked USP11 removes both Ub-K48 and Ub-K63 linkages on tau and enhances deubiquitination of Tau at K281. This deubiquitination facilitates enzymatic regulation of K281/K274 tau acetylation, which serves as a molecular switch leading to tau phosphorylation at S262 and subsequent pathological tau deposition [63, 64]. Genetic elimination of USP11 in a tauopathy mouse model showed preferential protection in females against acetylated tau accumulation, tau pathology, and cognitive impairment [63]. Garcia et al. also support the view that USP11 may contribute to the higher incidence in females of Alzheimer’s disease by combining analysis of single-cell datasets and publicly available bulk transcriptomics datasets [65]. These findings shed light on the higher incidence of AD in females and suggest that inhibiting USP11 could offer a promising therapeutic strategy to protect women from increased vulnerability to AD and other tauopathies.

### DUBs regulate Aβ and Tau

#### Overexpression of UCH-L1 accelerated the degradation of BACE1 and Tau

UCH-L1 is crucial for normal synaptic structure and function in mammalian hippocampal neurons [66]. Dysfunction of UCH-L1 has been shown to be strongly associated with human NDs [67]. In the context of AD, UCH-L1 has been shown to interact with APP and regulate Aβ production, and low UCH-L1 expression may be partly responsible for the pathophysiology and cognitive impairment in AD [68]. Decreased levels of soluble UCH-L1 protein were detected in both AD patients and APP/PS1 mouse models [69]. It increases free ubiquitin levels and promotes the ubiquitination and lysosomal degradation of APP, thereby reducing Aβ levels. Studies have revealed decreased levels of soluble UCH-L1 protein in AD patients and AD mouse models [67]. Gong et al. showed that the introduction of UCH-L1 rescued the synaptic and cognitive function of AD model mice, the therapeutic effect may depend on UCH-L1 deubiquitinating activity, because C90S mutant did not show any significant effect [69]. Furthermore, UCH-L1 inhibition increased the level of the β-secretase enzyme BACE1 and reduced the level of the subsequent BACE1 cleavage product Aβ. Overexpression of UCH-L1 accelerated the degradation of BACE1 in HUCH cells, a UCHL1 stably over-expressed HEK293 cell line [68].

p-Tau is mainly component of NFTs, which is an obvious pathological feature of AD, and soluble UCH-L1 is inversely proportional to the number of NFTs in AD brains [70]. In SH-SY5Y cells, UCH-L1 expression level also influences the phosphorylation of Tau protein. Inhibition of UCH-L1 increased the level of p-Tau while UCH-L1 overexpression decreased the level of p-Tau. Modulating UCH-L1 to reduce p-Tau levels could contribute to AD treatment [71].

Oxidative stress is recognized as a key factor in the pathogenesis of several age-related neurodegenerative diseases, including PD and AD [50, 72]. The UCH-L1 S18Y mutation has been associated with a lower incidence of PD, partly due to increased antioxidant capacity in neuronal cells expressing the UCH-L1 S18Y variant [73–75]. However, this protective effect against PD is not observed in AD [76]. Increased levels of oxidatively modified UCH-L1 have been reported in the brains of AD patients compared to normal individuals [77]. Several methionine and cysteine residues of UCH-L1 have been identified as potential targets for oxidation [70]. Oxidative modification of UCH-L1 and subsequent reduction in enzyme activity may impact neuronal function and survival, contributing to the pathogenesis of AD and PD [78]. Consequently, overexpressing UCH-L1 in the brain may represent a promising strategy for mitigating AD [67].

#### UCH-L3 hydrolyzes the UBB^+1^ C-terminus in HEK293 cells

In addition to UCH-L1, UCH-L3 and UCH-L5 have also emerged as potential regulators of AD. UCH-L3 is implicated in spatial and working memory [79]. In patients with AD, a mutant form of ubiquitin called UBB^+1^, characterized by a 19-amino acid extension, coexists with neuritic plaques and neurofibrillary tangles [80]. This UBB^+1^ protein accumulates abnormally only in diseased brains [81]. Functionally, UBB^+1^ has been shown to inhibit the UPS, which is closely related to the pathogenesis of AD [82]. When HEK293 cells were transiently co-transfected with UBB^+1^and UCH-L3. Cleavage of UBB^+1^ C-terminal is strikingly increased, generating a truncated form of UBB^+1^. However, oxidized UCH-L3 loses its ability to cleave UBB^+1^ [83]. Both full-length and truncated UBB^+1^ have demonstrated the ability to suppress the UPS, suggesting that the relationship between UCH-L3 oxidation and AD requires further investigation.

#### UCH-L5 regulates the development of central nervous system

UCH-L5 is associated with the 19S regulatory subunit of the 26S proteasome and plays a role in cleaving polyubiquitin chains from target proteins [84]. Deletion of UCH-L5 leads to severe defects in the development of the embryonic central nervous system, resulting in prenatal death in mice [85]. UCH-L5 is also identified as a hub connecting the proteasome-associated module with the histone acetyltransferase-associated module [86]. Downregulation of UCH-L5 may disrupt the coordination between transcriptional regulation and protein degradation.

#### USP13 Depletion is beneficial to p-Tau reduction and Aβ ubiquitination

Liu et al [87]. observed a twofold increase in USP13 levels in postmortem AD brains. Knockout of USP13 significantly enhanced the activity of the 20S proteasome and reduced the expression level of p-Tau in mouse primary cortical neuronal cells. These findings suggest that inhibiting USP13 may represent a therapeutic strategy to decrease plaque and toxic p-Tau accumulation in AD and other human tauopathies [88].

Furthermore, in animal models with USP13 knockdown, Aβ clearance was achieved by increasing the activity of the E3 ubiquitin ligase parkin [89, 90]. Collectively, these findings highlight the potential of targeting USP13 as a therapeutic approach for neurodegenerative diseases.

## Development inhibitors targeted on DUBs associated with AD

As mentioned above, some DUBs promote the onset and progression of AD by regulating autophagy, proteasome degradation, endoplasmic reticulum stress, etc. suggesting that DUB inhibitors hold significant potential in the treatment of AD and other neurodegenerative disorders. Based on their specific catalytic pockets, companies have developed a series of small molecule inhibitors (Table 2). However, it is worth noting that while DUB inhibitors have primarily been utilized in the field of tumor therapeutics, their application in NDs is still in its nascent stages [19]. Here, we specifically listed several currently known DUB small molecule inhibitors that have been used in AD Preclinical trials (Table 3).

**Table 2:** Inhibitors targeted on the AD-related DUBs.

Target	Compounds	Refs.
USP8	DUBs-IN series	[91–94]
	DC-U4106	[95]
	USP8-IN series	CN111138358A
	OTUB1/USP8-IN-1	[96]
USP25	AZ series	[40, 152]
	FT206	WO2020033707
	Vismodegib	[100]
	CT series	[101]
	C44	[102]
	miR-27a-3p	[103]
USP9	EOAI3402143	[109]
	FT709	[112]
	Degrasyn	[108]
OTUB1	OTUB1/USP8-IN-1	[96]
	Ubv.B1.1	[116]
	AVT	[114]
	miR-542-3p	[115]
USP14	IU1 series	[59, 121, 122, 153]
	CID 43013232	[123]
	CID 112370349	[123]
	b-AP15	[119]
	VLX1570	[117, 120]
USP10	Spautin-1	[125]
	P22077	[126]
	HBX19818	[126]
	Wu-5	[127]
	Quercetin	[128]
	CNYP	[129]
	UbV.10.1	[130]
USP11	Sennoside A, B	[131]
	MTX	[131]
	Tetrachloroisophthalonitrile	[131]
	Epirubicin	[131]
	Rutoside	[131]
UCH-L1	LDN-57444	[154, 155]
	LDN-91946	[137]
	6RK73	[140]
	8RK59	[141]
	8RK64	[141]
	IMP‑1710	[142]
	Z-VAE(OMe)-FMK	[145]
	GK13S	[146]
Broad-spectrum	PR-619	[147]
DUB inhibitor	RA-9	[149, 150]
	RA-14	[149]
	AM146	[149]

**Table 3:** Therapeutic effect of inhibitors of the DUBs targeting on AD.

Compounds	Structure	Target	Functions	R & D stage	Refs.
AZ1		USP25	Reduces the amount of APP and amyloid plaques	Preclinical trial	[40, 152]
IU1		USP14	Enhances Tau and TDP-43 degradation	Preclinical trial	[59, 153]
LDN-57444		UCH-L1	Decreases microtubule binding ability of Tau protein, increased phosphorylation level and abnormal aggregation	Preclinical trial	[67, 154, 155]
PR-619		Broad-spectrum DUB inhibitor	Leads to dephosphorylation of Tau	Preclinical trial	[156]

### Inhibitors targeting USP8

In 2010, A group led by Colombo identified 9-oxo-9H-indeno[1,2-b] pyrazine-2,3-dicarbonitrile as an active inhibitor of USPs, which are hydrolytic enzymes involved in the removal of ubiquitin from protein substrates [91]. Subsequent analogues of this compound, such as DUBs-IN-1, DUBs-IN-2 (HBX90659), and DUBs-IN-3, demonstrated potent inhibition of USP8 without affecting USP7 activity [91]. These inhibitors have primarily been studied in cancer research, where they have shown efficacy in reducing the viability of colon, lung, and prostate cancer cell lines by inhibiting USP8 [91–94]. Estrogen receptor (ER) is overexpressed in more than 70% of breast cancer patients, ERα is a classic estrogen receptor subtype, which can activate the oncogenes and induce the occurrence of breast cancer. A novel USP8 inhibitor, DC-U4106 could specifically inhibit USP8 and facilitate the degradation of ERα [95].

Additionally, Yucheng et al. developed a USP8-targeted small molecule regulator with a thiourea structure as the core, leading to the discovery of a series of high-activity compounds known as USP8-IN inhibitors (CN111138358A). Both OTUB1 and USP8 had been reported to promote tumorigenesis in NSCLC cells, in non-small-cell lung cancer (NSCLC) cell lines, OTUB1/USP8-IN-1 is a potent dual inhibitor of OTUB1 and USP8, reduced their substrate levels of the ubiquitin-conjugating enzyme E2 N (UBE2N) and EGFR [96], and then inhibit the proliferation of NSCLC cells.

### Inhibitors targeting USP25

A small-molecule discovery campaign based on the ubiquitin-rhodamine cleavable assay identified a panel of compounds with a thienopyridine carboxamide scaffold that selectively inhibited USP28 and USP25 [97, 98]. One such derivative, FT206, exhibited optimal drug metabolism and pharmacokinetic properties while preserving potency and selectivity towards USP28/25, which resulted in a dramatic decrease in c- MYC, c- JUN, and Δp63 proteins levels and consequently induced substantial regression of autochthonous murine LSCC tumors and human LSCC xenografts [98]. In addition, a series of benzylaminoethanols represented by AZ1, AZ2, AZ3and AZ4 were identified as strong inhibitors against USP28/USP25 during the validation phase of AZ series inhibitors [98]. Among them, AZ1 has been shown to inhibit USP25 and attenuated Wnt and SOCS3-PStat3 signaling, thereby promoting clearance of infected bacteria and resolution of inflammation, which inhibited colonic tumorigenesis [99]. Furthermore, Vismodegib, an FDA-approved inhibitor of the Hedgehog signaling pathway used for treating basal cell carcinoma, has also demonstrated inhibitory activity against USP25 (IC50: 1.42 μM) and USP28 (IC50: 4.41 μM) [100].

To identify inhibitors of USP25 and USP28, Peng et al. screened a library of 100,000 synthetic compounds and identified three lead compounds, CT1001-1003, which displayed significant inhibitory activity [101]. Further optimization of these compounds led to CT1113, it is a potent USP28 and USP25 inhibitor, experiments demonstrate that CT1113 can significantly increase the general level of USP25 substrate Tankyrase (TNKS) and inhibit the proliferation of various tumor cells, including Pancreatic Cancer Cancel LINE SW1990, Colon Cancer Cell Line HCT116 and lung cancer A549, etc., showing broad anti-tumor activity [101]. Using a hierarchical virtual screening approach, Cheng et al [102]. identified a small molecule called C44 that specifically binds to the protein-protein interaction (PPI) interface of TNKS and USP25, disrupting their interaction and leading to a higher half-life of AXIN and the breakdown of β-catenin protein, significantly attenuates prostate cancer cell proliferation.

Additionally, miR-27a-3p, a highly conserved non-coding RNA, has been found to regulate USP25 and USP46 expression by binding to its 3’-untranslated region [103, 104]. Downregulation of USP25 by miR-27a-3p contributes to the inhibition of trophoblast cell migration and invasion through the epithelial-to-mesenchymal transition (EMT) process [103].

### Inhibitors targeting USP9

Degrasyn (WP1130) is an inhibitor that can penetrate cells, which directly inhibits the DUB activity of USP9X, USP5, USP14, and UCH37 [105–107]. It has demonstrated the ability to downregulate antiapoptotic proteins such as Mcl-1, Bcr-Abl and JAK2, resulting in the apoptosis of myeloid and lymphoid tumor cells, but as time goes by, the inhibitory of MCL-1 has weakened [108]. On this basis, Peterson et al. improved the drug-like properties of WP1130 and demonstrated that the novel compound EOAI3402143, which dose-dependently inhibits USP9X, USP5, and USP24, increasing tumor cell apoptosis [109–111]. FT709 is a potent and selective inhibitor of USP9X, with an IC50 value of 82 nM [112]. It has been linked to various cellular processes such as centrosome function, chromosome alignment during mitosis, EGF receptor degradation, chemo-sensitization, and circadian rhythms by inhibiting USP9X [112].

### Inhibitors target OTUB1

Many of the OTUB1 inhibitors currently used in research are spectral inhibitors, including PR-619 and N-ethylmaleimide [113]. OTUB1/USP8-IN-1 is a potent dual inhibitor of OTUB1 and USP8, which inhibits the development of NSCLC cells, as previously mentioned [96]. Acevaltrate (AVT), derived from Valeriana glechomifolia, was identified as an inhibitor based on its activity against the OTUB1/c-Maf/luciferase system. AVT disrupts the interaction between OTUB1 and the oncogenic transcript factor c-Maf, leading to c-Maf proteasome degradation and induced myeloma cell apoptosis [114]. miR-542-3p has been identified as a tumor suppressor in multiple cancer types. Overexpression of miR-542-3p significantly reduces OTUB1 mRNA and protein levels, inhibiting migration and invasion of esophageal cancer cells [115].

To explore inhibitors with specificity for OTUB1, researchers synthesized and screened ubiquitin point mutants. Ubv.B1.1 was identified as a ubiquitin mutant that binds to the distal ubiquitin binding site of OTUB1, effectively inhibiting its catalytic activity and interfering with OTUB1-E2 complex formation [116].

### Inhibitors targeting USP14

**Fig. 3:**
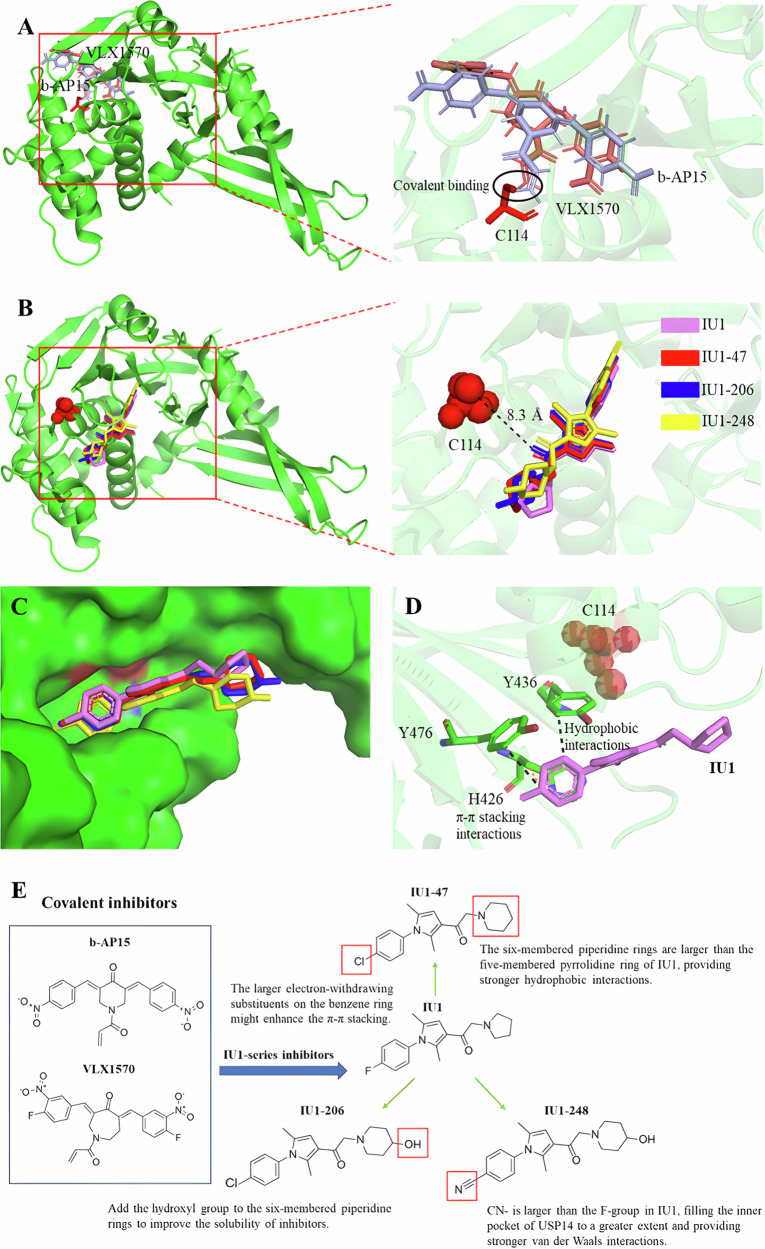
Structural basis for development inhibitors specifically targeted on USP14. **A** Covalent binding of b-AP15 and VLX1570 to the active center C114 of USP14. **B** Co-crystal structure of USP14 in complex with IU1-series inhibitors, illustrating the allosteric inhibition mode. The IU1-series compounds share a similar binding pocket located 8.3 Å away from the catalytic center C114. **C** Binding pocket for IU1-series inhibitors in USP14. **D** Detailed view of the binding pocket in (**C**), highlighting the residues of USP14 that interact with IU1. The benzene ring of IU1 engages in hydrophobic interactions and π-π stacking with H426, Y436, and Y476 residues. **E** The development of USP14 inhibitors from covalent inhibitors to structure-guided optimization of allosteric inhibitors. The development of USP14 inhibitors has gradually shifted from covalent inhibitors to allosteric inhibitors. Based on the structural analysis of the essential interactions between the allosteric inhibitor IU1 and USP14, the optimization of IU1 was performed. On one hand, the inclusion of electron-withdrawing substitutes on the ozone ring were explored, which might enhance the π-π stacking. On the other hand, ever different rings were used to replace the pyrrolidine ring, which extended into the solvent-exposed region. In Fig. 3, USP14 is depicted as a surface model, with the catalytic center represented as a sphere model. IU1-series inhibitors are shown in stick model. IU1, IU47, IU1-206, and IU1-248 are shown in violet, red, blue, and yellow, respectively. The dotted line represents hydrophobic interactions, while orange dotted lines are π-π stacking interactions.

USP14 is a ubiquitin-specific protease that is associated with the proteasome and plays important roles in cellular functions, viral infection, inflammatory responses, neurodegenerative diseases, and tumorigenesis [117]. The important roles of USP14 in multiple diseases have encouraged the development of clinically viable USP14 antagonists, so scientists have made a lot of efforts, from covalent inhibitor to allosteric inhibitor, USP14 has become a relatively mature candidate for the development of small molecule inhibitors of AD-related DUBs, the development, structure, and optimization process of USP14 small molecule inhibitors are shown here (Fig. 3).

b-AP15 has demonstrated inhibitory activity against both USP14 and UCHL5, two deubiquitinating enzymes associated with the 19S regulatory particle, in a covalent manner [118]. It has been shown to inhibit tumor progression in four different in vivo solid tumor models and inhibited organ infiltration in an acute myeloid leukemia model by inhibiting USP14 and UCHL5 [119]. Its analog, VLX1570, possesses improved potency and enhanced solubility. VLX1570 interacts with the critical catalytic cysteine residues located at the active center of both USP14 and UCHL5 (Fig. 3A). Unfortunately, clinical research on VLX1570 for multiple myeloma treatment was discontinued due to severe lung toxicity [105, 106]. The therapeutic potential of these covalent inhibitors for other DUB enzymes, such as UCHL5, remains uncertain due to their poor selectivity [117, 120].

Consequently, researchers have shifted their focus to the development of allosteric inhibitors for USP14. The group led by Finley reported the first specific USP14 inhibitor, named IU1 [121]. Subsequently, derivatives of IU1 have been synthesized, including IU1-206 and IU1-248 and IU1-47, which are potent and selective inhibitors of USP14 with an IC50 value 10 times more potent than IU1 [121, 122]. High-resolution co-crystal structures of IU1-series inhibitors and USP14 have been resolved, revealing that these compounds share a similar binding pocket located 8.3 Å away from the catalytic center, acting as allosteric inhibitors (Fig. 3B, C) [117]. The benzene ring of IU1 engages in π-π stacking and hydrophobic interactions with the H426, Y436, and Y476 residues of USP14. The perpendicular orientation of the benzene ring to the pyrrole ring reinforces its interaction with H426, Y436, and Y476 residues. The two methyl groups of the pyrrole ring are also crucial for the inhibitory capacity of IU1, contributing to π-π stacking (Fig. 3D). Due to the importance of the benzene ring and the dimethyl-substituted pyrrole ring for π-π stacking, this main skeleton was maintained during the optimization of the IU1-series inhibitors. To enhance π-π stacking, researchers have attempted to replace the F- group on the benzene ring of IU1 with other electron-withdrawing substitutes (e.g., Cl- in IU1-47, IU1-206, and CN- in IU1-248). Moreover, larger rings and polar groups have been incorporated to replace the pyrrolidine rings, extending into the solvent-exposed region, to improve binding affinity and compound solubility. For example, in IU1-47, the five-membered pyrrolidine ring of IU1 is replaced by a six-membered piperidine ring, providing stronger hydrophobic interactions. In IU1-206 and IU1-248, the hydroxyl group is attached to the six-membered piperidine ring to improve solubility (Fig.3E). Following optimization, both IU1-47 and IU1-248 exhibit higher selectivity for USP14. Notably, IU1-248 demonstrates significantly improved solubility compared to IU1, which may be advantageous in cell-level studies [117].

Using the high-resolution crystal structure as a guide, researchers have identified potential allosteric inhibitors of USP14 through screening a compound library with chemical structures analogous to IU1 [123]. Among them, CID43013232 and CID112370349 have demonstrated better binding affinity than IU1 [123].

### Inhibitors targeting USP10

Spautin-1 is a specific and potent autophagy inhibitor that inhibits both USP10 and USP13 [124]. spautin-1 enhances imatinib mesylate-induced apoptosis by inactivating PI3K/AKT and activating downstream GSK3β, leading to downregulation of Mcl-1 and Bcl-2, which inhibited autophagy and enhanced apoptosis in chronic myeloid leukemia cells [125]. However, Spautin-1 cannot effectively cross the blood-brain barrier, which poses a major challenge for inhibiting USP10 in the treatment of Alzheimer’s disease (AD) [90]. P22077 and HBX19818, initially identified as irreversible USP7 inhibitors, may also inhibit the deubiquitinating activity of USP10, thereby preventing the proliferation of FLT3-ITD-positive tumor cells [126]. Wu-5, a novel USP10 inhibitor identified through compound library screening, inhibits the proliferation of MV4-11 cells primarily by inhibiting USP10 activity and subsequently reducing the expression of the downstream gene AMPKa [127].

Quercetin is a pentahydroxyflavone found in various fruits and vegetables, which has been shown to reduce the expression of USP10. It interacts with USP10 and contributes to the degradation of T-bet, resulting in inflammation reduction [128]. In 2009, Cai’s Neiyi Prescription (CNYP), a medicine invented by Cai, exhibits anti-inflammatory properties by inducing the apoptosis of endometrial stromal cells. CNYP has been found to reduce mRNA and protein expression of USP10 [129]. Unlike other USP10 inhibitors, UbV.10.1 is a mixture of proteins or peptides that possess high affinity for USP10 and inhibit its activity. It was identified through screening a phage-displayed ubiquitin variant (UbV) library and considered an inhibitor of endogenous USP10 in cells [130].

### Inhibitors targeting USP11

A systematic high-throughput approach was employed to screen 2,000 U.S. FDA-approved compounds for their ability to inhibit USP11 enzymatic activity [131]. Six pharmacologically active small molecules were identified as inhibitors of USP11, including Sennoside A, Sennoside B, Mitoxantrone (MTX), Tetrachloroisophthalonitrile, Epirubicin, and Rutoside. Among them, MTX demonstrated an impact on pancreatic ductal adenocarcinoma (PDA) cell survival with an IC50 of less than 10 nM [131]. Prior reports have documented USP11 works in concert with BRCA2 to facilitate DNA homologous recombination by recruiting components of the DNA repair complex, MTX targets USP11 and inhibits pancreatic cancer cell survival [132]. In addition, MTX also exhibits inhibitory effects on the USP11 homolog, USP15 [133].

### Inhibitors targeting UCH-L1

LDN-57444 is a reversible, competitive, and site-directed inhibitor of UCH-L1 and UCH-L3 [134]. UCHL1 binds, deubiquitinates, and stabilizes POM121 to regulate POM121-associated nuclear transport of E2F1 and c-MYC. Treatment with the UCHL1 inhibitor LDN-57444 slows tumor growth and metastasis across neuroendocrine carcinomas such as neuroendocrine prostate cancer and small-cell lung cancer [135]. However, recent investigations have indicated that LDN-57444 exhibits off-target toxicity and chemical instability [136]. In contrast, its derivative, LDN-91946, has shown potent, selective, and uncompetitive inhibition of UCH-L1, with no cytotoxicity of serum-starved Neuro 2 A (N2A) cells at concentrations as high as 0.1 mM [137]. It also attenuated the proliferation of both lung cancer and mesothelioma cell lines [138].

In recent years, small-molecule covalent inhibitors of UCH-L1 containing a cyanopyrrolidine moiety have been reported in patent literature like MT16-001 and MT16-205, but these two compounds failed to inhibit UCH-L3 which shares 51% sequence identity with UCH-L1 [139]. 6RK73 is a covalent irreversible and specific UCHL1 inhibitor which shows almost no inhibition of UCHL3 [140]. UCHL1 facilitates TGFβ signaling-induced metastasis by protecting TGFβ type I receptor and SMAD2 from ubiquitination, 6RK73 antagonizes TGFβ/SMAD signaling and inhibits breast cancer migration and extravasation [140]. Various fluorescent small-molecule activity-based probes (ABPs) have been designed based on the cyanopyrrolidine inhibitor 6RK73 coupled with different fluorescent reporters. One well-characterized example is the BodipyFL probe 8RK59 [141]. Additionally, Panyain et al. have reported a series of small molecule inhibitors, including IMP-1710, based on a Cyanopyrrolidine-based UCH-L1 inhibitor. IMP-1710 a potent and selective UCH-L1 inhibitor which labels C90 of UCH-L1 at nanomolar concentrations in a cell model of idiopathic pulmonary fibrosis, blocking the profibrotic response [142].

**Fig. 4:**
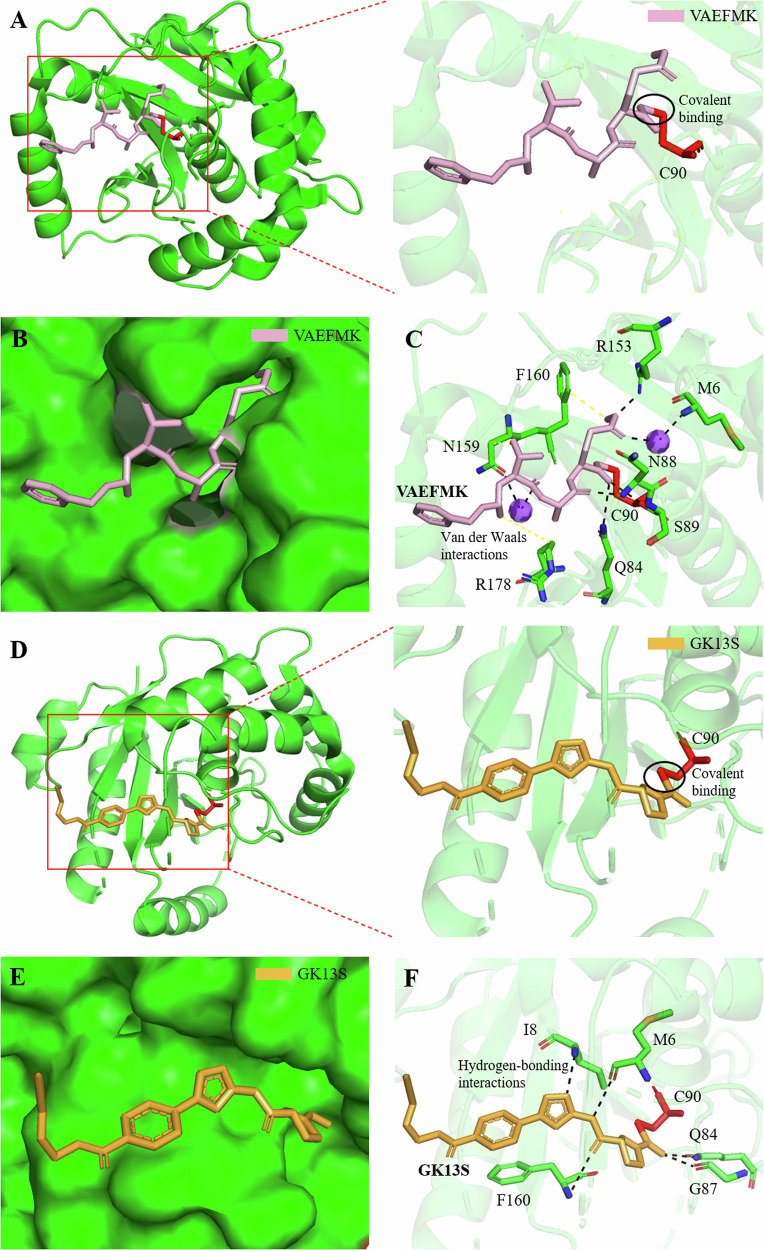
Structural basis for inhibitors of UCH-L1. **A** Covalent binding of Z-VAE(OMe)-FMK (VAEFMK) to the active center C90 of UCH-L1. **B** Binding pocket for VAEFMK in UCH-L1. **C** Interactions stabilizing VAEFMK within UCH-L1’s active site. VAEFMK forms a covalent bond with the catalytic center C90, while surrounding residues establish hydrogen bonding and van der Waals interactions to stabilize VAEFMK. **D** Covalent binding of GK13S to the active center C90 of UCH-L1. **E** Binding pocket for GK13S in UCH-L1. **F** nteractions stabilizing GK13S within UCH-L1’s active site. GK13S covalently binds to the catalytic center C90, and nearby residues (M6, I8, Q84, G87, and F160) contribute to stabilizing GK13S through hydrogen bonding. In Fig. 4, UCH-L1 is depicted as a surface model, with the catalytic center represented as a sphere model. The inhibitors GK13S and VAEFMK are shown in stick model, colored in orange and magenta, respectively. Black dotted lines represent hydrogen-bonding interactions, while yellow dotted lines represent van der Waals interactions.

Cysteine proteases can be covalently inactivated by peptide halomethyl ketones [143]. Fluoromethylketones (FMKs) are irreversible inhibitors of cysteine proteases, as they alkylate the active-site thiol, resulting in the displacement of the halide group by the catalytic cysteine to form a thioether bond between the cysteine and the inhibitor [144]. The X-ray co-crystal structure of UCH-L1 with Z-VAE(OMe)-FMK (benzyloxycarbonyl-Val-Ala-Glu(γ-methoxy) fluoromethylketone) at 2.35 Å resolution reveals that the interaction is primarily maintained by hydrogen bonds between the groups constituting the oxyanion hole of UCH-L1 and the backbone of VAEFMK (Fig. 4A, B) For example, the backbone NH groups of S89 and C90 form hydrogen bonds with the backbone carbonyl oxygen atom of the Glu (OMe) residue of the inhibitor. The carbonyl group of the Ala residue of the inhibitor is within hydrogen-bonding distance of the backbone NH groups of N88 and the side chain NH2 group of the oxyanion-stabilizing residue, Q84. In addition to these hydrogen-bonding interactions, van der Waals interactions also contribute to stabilizing the bound inhibitor. For instance, the Cβ atom of the alanine residue in the inhibitor contacts the side chain of R178, which appears to have rotated relative to its position in the native structure to accommodate the inhibitor. Furthermore, the hydrophobic side chain of Val in VAEFMK is nestled against the side chain of N159. In one of the subunits, the inhibitor appears to be better defined within the active site, possibly due to additional interactions with two protein-bound water molecules in the active site. The backbone NH of M6 forms a hydrogen bond with a water molecule, which, in turn, hydrogen bonds with the carbonyl group on the side chain of the Glu (OMe) residue. Another water molecule is held in place by hydrogen bonding with the side chain of N159 (Fig. 4C). This binding mode may be advantageous from the perspective of specificity [145].

However, inhibitors of UCH-L1 are still limited and lack specificity or cytotoxicity. In 2022, Grethe et al. reported the development of potent and non-toxic chemogenomic activity-based probes GK13S and GK16S for UCH-L1 [146]. They contain an Alkyne group and can undergo copper-catalyzed azide-alkyne cycloaddition (CuAAc) with molecules containing Azide groups [146]. Biochemical characterization of GK13S demonstrated its stereoselective inhibition of cellular UCH-L1. The crystal structure of UCH-L1 in complex with GK13S revealed that side chains of certain residues of UCH-L1 form a narrow hydrophobic pocket surrounding the pyrrolidine moiety of GK13S, stabilizing its binding around the active center (Fig. 4D, E). GK13S covalently binds to the catalytic cysteine C90 of UCH-L1 through an isothiourea with a cyanamide warhead. The isothiourea is stabilized by the oxyanion hole formed by N84 and G87, with the catalytic triad residues in hydrogen bonding distance (Fig. 4D). The central amide and imidazole ring of GK13S engage with UCH-L1 on both sides of the cleft, forming hydrogen bonds with the backbone of F160, M6, and I8 (Fig. 4F). Phenocopying a reported inactivating mutation of UCH-L1 in mice, GK13S, but not GK16S, leads to reduced levels of monoubiquitin in a human glioblastoma cell line [146].

### Broad-spectrum DUB inhibitors

2,6-Diaminopyridine-3,5-BIS (thiocyanate) (PR-619) is a broad-spectrum DUB inhibitor [147]. MS analysis revealed that PR-619 interferes with the labeling of several DUBs, including USP1, 4, 8, 10, 16, 19, 22, 24, 28, 48, VCIP135, OTUD5, BAP1, ATXN3, YOD1, and UCH-L5 [148]. It effectively enhanced the cisplatin-induced antitumor effect via concurrent suppression of the Bcl-2 level in metastatic bladder urothelial carcinoma (T24 and BFTC-905) cells [147].

Chalcones exhibit selective anti-neoplastic activity by inhibiting the 26S proteasome activity. Chalcone derivatives, such as AM146, RA-9, and RA-14, have demonstrated anticancer activity by targeting 19S RP associated DUBs, including USP2, USP5, USP8, USP14, UCH-L1, UCH-L3, and UCH37, without affecting the catalytic core activity of the 20S proteasome [149, 150]. Among them, RA-9 as a small-molecule inhibitor of proteasome-associated DUBs like USP14 and UCHL-37, has been shown to selectively induce onset of apoptosis in ovarian cancer cell lines and primary cultures derived from donors [150].

N-Ethylmaleimide (NEM), derived from maleic acid, can alkylate free sulfhydryl groups and irreversibly inhibit the catalytic activity of a broad range of cysteine-dependent DUBs [151]. Studies have shown that NEM blocked platelet-derived growth factor-BB-induced Akt phosphorylation without affecting its upstream PI3K, interfered with the PI3K/Akt pathway at the level of Akt. participating in redox regulation of Akt and cell survival [151].

## Therapeutic effect of inhibitors targeted on AD-associated DUBs

Although several inhibitors targeting AD-related DUBs have been developed as described above, their suitability for AD treatment is currently limited due to cytotoxicity or inability to cross the blood-brain barrier. Only a few relatively advanced inhibitors have reached the preclinical stage (Table 3). It is hoped that more effective inhibitors will be developed and applied in the future for the treatment of AD.

### AZ1

AZ1 has shown potential in ameliorating AD neuropathology by inhibiting USP25 and attenuating microglial activation [40]. In a recent study, transgenic mice with familial Alzheimer’s disease (5×FAD mice) treated with AZ1 showed significant reductions in the amount of APP and amyloid plaques in the cerebral cortex, suggesting that AZ1 improved amyloid overload in an AD mouse model through pharmacological inhibition of USP25 [152].

### IU1

A small molecule inhibitor called IU1 has been shown to enhance proteasomal substrate degradation in mouse embryonic fibroblasts overexpressing Tau or TAR DNA-binding protein-43 (TDP-43) (Fig. 3A) [57]. Interestingly, IU1 treatment had no effect on proteasome degradation of Tau in neurons, suggesting that degradation of Tau may be mediated by calpain rather than the 26S proteasome [153]. Inhibition of USP14 has been proposed as a therapeutic strategy to enhance proteasomal function in AD [57, 59]. However, IU1 has been found to be neurotoxic even at low concentrations, which may hinder its application as a drug for AD treatment [59].

Additionally, IU1-47 has been shown to stimulate Tau degradation primarily through the ubiquitin-proteasome system, leading to significant reductions in Tau and p-Tau Ser-202/Thr-205 levels in murine cortical primary neurons [122].

### LDN-57444

LDN-57444, a competitive and active site-directed inhibitor of UCH-L1 with 28-fold greater selectivity over UCH-L3, has been studied in the context of AD [69]. Research has demonstrated that the inhibition of UCH-L1 by LDN-57444 leads to significant accumulation of APP protein and continuous accumulation of BACE1 protein [67, 68]. These findings suggest that UCH-L1 plays a crucial role in promoting the degradation of Aβ. Moreover, UCH-L1 is negatively correlated with neurofibrillary tangles (NFTs) in the AD brain. Since NFTs mainly consist of p-Tau protein, inhibition of UCH-L1 activity by LDN-57444 may decrease the microtubule-binding ability of Tau, leading to increased phosphorylation levels and abnormal aggregation [154, 155].

### PR-619

Treatment with PR-619 has been shown to lead to dephosphorylation of Tau and promote the association of Tau with microtubules, suggesting a potential role in controlling AD and other neurodegenerative diseases through Tau phosphorylation [156]. However, as PR-619 is a pan-inhibitor of DUBs, it remains unclear which specific DUBs are responsible for these physiological responses.

## Conclusion and perspective

The global incidence of Alzheimer’s disease (AD) is steadily increasing, and neurodegenerative diseases (NDs) are characterized by the accumulation of toxic proteins. The ubiquitin-proteasome system (UPS) is responsible for the degradation of most proteins in humans, and enzymes involved in this process are often important regulators of disease pathogenesis and attractive targets. Among them, deubiquitinating enzymes (DUBs) have emerged as potential therapeutic targets in NDs. Several DUBs, including USP8, USP25, USP14, and UCH-L1, have been implicated in the physiological processes of AD. However, our understanding of DUBs in AD is still limited, and there are intriguing aspects that require further exploration.

Firstly, it is essential to identify new DUBs associated with AD. Currently, only a few DUBs have been linked to AD, which is insufficient to fully explore the underlying pathogenesis and potential treatments. Moreover, while current studies primarily focus on AD and Parkinson’s disease (PD), the association of DUBs with other NDs such as Huntington’s disease (HD), amyotrophic lateral sclerosis (ALS), and frontotemporal dementia (FTD) remains largely unknown. Therefore, concerted efforts are needed to identify novel DUBs involved in different types of NDs.

Secondly, the exact mechanisms by which pathogenic proteins contribute to disease pathogenesis and how deubiquitinating enzymes regulate the levels of toxic proteins are not well understood. For instance, UCH-L1, USP8, USP9X, and USP13 have been implicated in regulating toxic proteins not only in AD but also in PD, suggesting potential involvement of DUBs in multiple degenerative brain diseases. Therefore, these DUBs hold promise for targeting toxic proteins associated with various neurodegenerative diseases, including Aβ, Tau, and α-synuclein. However, further investigation is needed to elucidate the precise mechanisms by which DUBs modulate the levels of toxic proteins. A clearer understanding of these regulatory mechanisms would facilitate the development of more effective strategies for the prevention and treatment of neurodegenerative diseases.

Thirdly, it is crucial to develop specific and potent DUB inhibitors. While DUBs play a crucial role in AD, only a limited number of DUB inhibitors have been discovered thus far. The existing small molecule inhibitors are primarily evaluated in cancer research, with limited application in AD studies. Moreover, most of the current inhibitors have certain limitations. For example, IU1 exhibits neurotoxicity even at low concentrations, which hinders its application in AD treatment. Spautin-1, an inhibitor of USP13 and USP10, may hold therapeutic potential for AD; however, its inability to cross the blood-brain barrier restricts its effectiveness. Notably, crystal structures of DUBs such as USP14 and UCH-L1 complexed with various inhibitors have provided insights into the allosteric or covalent regulatory mechanisms. These findings pave the way for the design of more potent inhibitors, offering potential for the discovery of targeted drugs for the treatment of AD.
